# A Clinico-Epidemiological Study of Various Autoimmune Vesiculobullous Disorders in a Tertiary Care Center

**DOI:** 10.7759/cureus.86571

**Published:** 2025-06-22

**Authors:** Vunnava Sri Koulini, Dilipchandra Chintada, Kirankanth Vudayana, Aruna Bathina

**Affiliations:** 1 Department of Dermatology, Venereology and Leprosy, Great Eastern Medical School and Hospital, Srikakulam, IND

**Keywords:** autoimmune vesiculobullous, corticosteroids, dcp therapy, intraepidermal, pemphigus vulgaris, rituximab, subepidermal

## Abstract

Background

Pemphigus and pemphigoid disorders, as well as dermatitis herpetiformis, are all part of the diverse group of conditions known as autoimmune vesiculobullous/blistering disorders (AIBDs). These disorders are caused by autoantibodies that target intercellular or cell-matrix adhesion proteins and are clinically characterized by blisters or erosions of the skin and/or mucous membranes. The incidence peaks at 30-60 years with an equal incidence in men and women. There are 0.1 to 0.5 cases of pemphigus for every 100,000 people.

Materials and methods

This single-center retrospective observational study was conducted at our tertiary care center (Great Eastern Medical School and Hospital) in Srikakulam, Andhra Pradesh, India, between March 2018 and March 2025, and examined 50 histopathologically and direct immunofluorescence confirmed cases of autoimmune vesiculobullous disorders over a seven-year period.

Results

The percentage of females, i.e., 28 (56%) out of 50 individuals, is marginally higher than that of males (22 (44%)). The most prevalent age group is 51-60 years old (26%), followed by 41-50 years old (20%). The majority of cases are from rural areas (72%) and are occupied by people from low socioeconomic backgrounds (72%). The majority of farmers are local residents (76%) with few migrants (24%). Of the 50 cases, the most prevalent intraepidermal variants were pemphigus vulgaris (18 cases) and pemphigus foliaceus (nine cases), with a single case of pemphigus vegetans, whereas the most prevalent sub-epidermal variants were bullous pemphigoid (14 cases), with a small number of epidermolysis bullosa acquisita cases (eight cases). In 44% of instances, mucosal involvement is observed. Comorbidities, such as diabetes, hypertension, heart disease, and malignancies, were found in a small percentage of cases. Corticosteroids were the first line of treatment, and immunosuppressants were used for maintenance. Few patients had positive outcomes using dexamethasone cyclophosphamide pulse therapy (DCP), and rituximab given in two cases showed disease control after the second dose. Better disease control was demonstrated by patients who adhered to the long-term treatment.

Conclusion

In recent years, autoimmune vesiculobullous illnesses have become more prevalent and are significantly correlated with other systemic conditions. A difficult problem that requires careful handling is atypical clinical manifestations, which can be linked to inappropriate and delayed treatment as well as noncompliance. Long-term steroid medication is less necessary with early diagnosis, which further minimizes consequences by evaluating the disease activity and severity and starting a targeted treatment. For the general improvement of quality of life, public education, development of medical services in rural regions, and the elimination of social stigma are, therefore, crucial.

## Introduction

Autoimmune vesiculobullous/blistering disorders (AIBDs) are a heterogeneous group of approximately a dozen entities comprising pemphigus and pemphigoid disorders and dermatitis herpetiformis [1], caused by autoantibodies that target intercellular or cell-matrix adhesion proteins [2]. These are characterized clinically by blisters or erosions of the skin and/or mucous membranes [3]. The peak incidence is between 30 and 60 years of age, and the mean age of onset is between 50 and 60 years. The prevalence in men and women is roughly equal [4].

Autoimmune bullous disorders are broadly classified into intraepidermal and subepidermal bullous disorders. The most common intraepidermal ABD is pemphigus, with a worldwide prevalence of 0.1-0.5 per 100,000 population, which can be as high as 3.2 per 100,000 in certain races [5].

The incidence of autoimmune bullous disorders has increased over the years, especially in elderly patients with multiple comorbidities, which has stimulated research into their association with other diseases [6].

Topical and systemic corticosteroids are the mainstays of initial treatment for bullous pemphigoid (BP) and pemphigus diseases. Additional immunomodulatory therapies such as methotrexate, azathioprine, and mycophenolate mofetil (MMF) should be added early during treatment to minimize the adverse effects of chronic corticosteroid therapy and to augment improvement in the disease. Rituximab is a first-line immunomodulatory treatment for moderate to severe pemphigus disease [7].

## Materials and methods

Study design

This single-center retrospective observational study was carried out from March 2018 to March 2025 at our tertiary care facility, Great Eastern Medical School and Hospital, in Srikakulam, Andhra Pradesh, India. The study included case records of patients from the northeast Andhra region with histological and direct immunofluorescence (DIF) evidence of autoimmune vesiculobullous illnesses after receiving institutional ethics committee ethical approval on March 20, 2025, with Reg. No. 13/IEC/GEMS&H/2025.

Study participants

The study included and examined case records of patients who were hospitalized in the Department of Dermatology, Venereology, and Leprosy with autoimmune vesiculobullous illnesses. The study includes patients of all ages, both sexes, histopathologically proven cases of autoimmune vesiculobullous disorders (with prior proven reports or evaluated and histopathology confirmed in our institute after admission), excluding histopathologically discordant cases, outpatient department (OPD) based cases, and cases with incomplete data from records. Fifty cases with histological confirmation were included in the study out of the 65 cases that came to our tertiary care facility.

Data collection

The comprehensive and detailed data included the following: information about age, gender, occupation, socioeconomic status, place of origin, history (complaints, onset), cutaneous examination details (skin lesions, mucosal, scalp involvement), bedside tests, systemic examination, laboratory investigations (routines, DIF, histopathology), and photographs of 50 cases of autoimmune vesiculobullous disorders were taken from our medical department institute record files.

The severity assessment was done by Autoimmune Bullous Skin Disorder Intensity Score (ABSIS) scoring, which includes skin involvement, mucosal involvement, and subjective discomfort. It ranges from 0 to 206 score, with higher scores indicating more severe disease. The severity levels can be graded as follows: limited pemphigus (score 0 to 3); moderate pemphigus (score 4 to 16); significant pemphigus (score 17 to 52); and extensive pemphigus (score 53 to 206).

After the data were collected, the results were tabulated and summarized.

Statistical analysis

Data were entered into Microsoft Excel (Microsoft® Corp., Redmond, WA) and analyzed using the OpenEpi version 3.01 (Dean AG, Sullivan KM, Soe MM, Emory University, Atlanta, GA) tool to provide a detailed and thorough summary of patient demographics, epidemiological, clinical, and histopathological features, and treatment outcomes. The association between a few categorical variables was analyzed using chi-square tests and considered to be significant when the p-value < 0.05.

## Results

Epidemiological data

Out of the 50 subjects, the proportion of females (28 (56%)) is slightly higher than that of the males (22 (44%)).

Overall data suggest a relatively higher distribution between the age group of 51 and 60 years, accounting for 26%, followed by 41-50 years, accounting for 20%, fewer individuals in extreme age groups, i.e., 0-10 years (2%) and 71-80 years (2%), with the youngest being nine years and the eldest being 79 years (Table 1). Rural population outweighed urban population, accounting for 72% and 28 % of the study population, respectively. Out of 50 cases, eight patients can read and write and understand the language, accounting for 16% of the literacy rate, while 42 are illiterate, accounting for 84%. A chi-square test showed no significant association between gender and type of disease (χ² = 0.00, p = 1.00) or between socio-economic status and disease type (χ² = 0.72, p = 0.395), as the p-value was <0.05.

**Table 1:** Distribution of participants by age group

Age group in years	Frequency	Percentage (%)
0-10	1	2
11-20	3	6
21-30	7	14
31-40	9	18
41-50	10	20
51-60	13	26
61-70	6	12
71-80	1	2
Total	50	100

**Figure 1:**
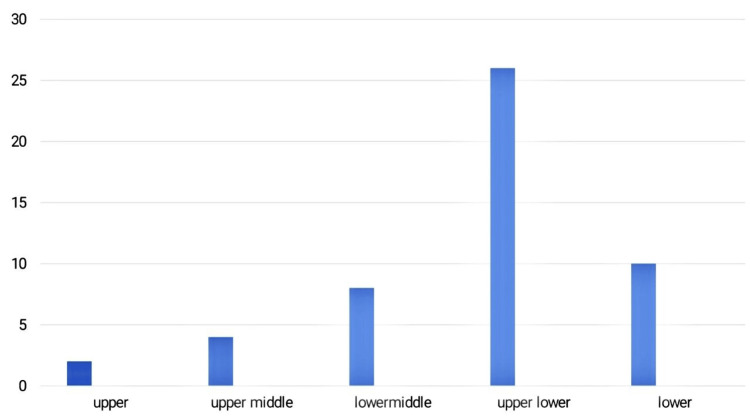
Bar chart showing distribution of 50 cases as per socio-economic status according to the modified Kuppuswamy scale The x-axis indicates the class of socioeconomic status. The y-axis indicates the number of cases.

Most of the patients, i.e., 36 (72%), belong to low socioeconomic status with agriculture being the most common occupation, while 24% of patients were of middle class, and 4% belonged to upper class according to the modified Kuppuswamy classification (Figure 1). Majority of patients are from local group, i.e., from North East Andhra districts comprising 20 (40%) from Srikakulam, seven (14%) from Vizianagaram, five (10%) from Visakhapatnam, six (12%) from Manyam districts while patients migrated from Orissa due to occupational purposes comprised 12 (24%) of the population (Table 2).

**Table 2:** Distribution of patients by region

Region	Area	Frequency	Percentage (%)
Migratory group	Odisha	12	24
Local group (North East Andhra)	Srikakulam	20	40
	Vizianagaram	7	14
	Vishakapatnam	5	10
	Manyam	6	12
Total		50	100

Clinical data

Autoimmune vesiculobullous disorders can be either intraepidermal or subepidermal types, and both have variations. The majority of the cases in this study were of intraepidermal type (28), of which the majority were of pemphigus vulgaris (PV) (18 (36%)), and 22 were of subepidermal type, of which the majority were of BP (14 (28%)) (Table 3). Nearly 75% of cases showed up within a year of the lesions starting, while only 15% did so within a year or two. The remaining cases showed up after two years. The majority of cases (86%) had vesicles and bullae with crusting (54%) and erosions (70%), as well as mucosal involvement (42%), and a small number of cases also had additional lesions like papules, erythema, and pustules (Table 4).

**Table 3:** Distribution of participants by different types of autoimmune vesiculobullous disorders

Type	Frequency	Percentage (%)
Pemphigus vulgaris	18	36
Pemphigus foliaceus	9	16
Pemphigus vegetans	1	2
Bullous pemphigoid	14	28
Epidermolysis bullosa acquisita	8	16

**Table 4:** Distribution of participants by various clinical presentations

Presentation	Frequency	Percentage (%)
Vesicles and bullae	43	86
Erosions	35	70
Crusting	27	54
Mucosal lesions	21	42
Papules	5	10
Pustules	8	16
Erythema	15	30
Scaling	7	14

**Figure 2:**
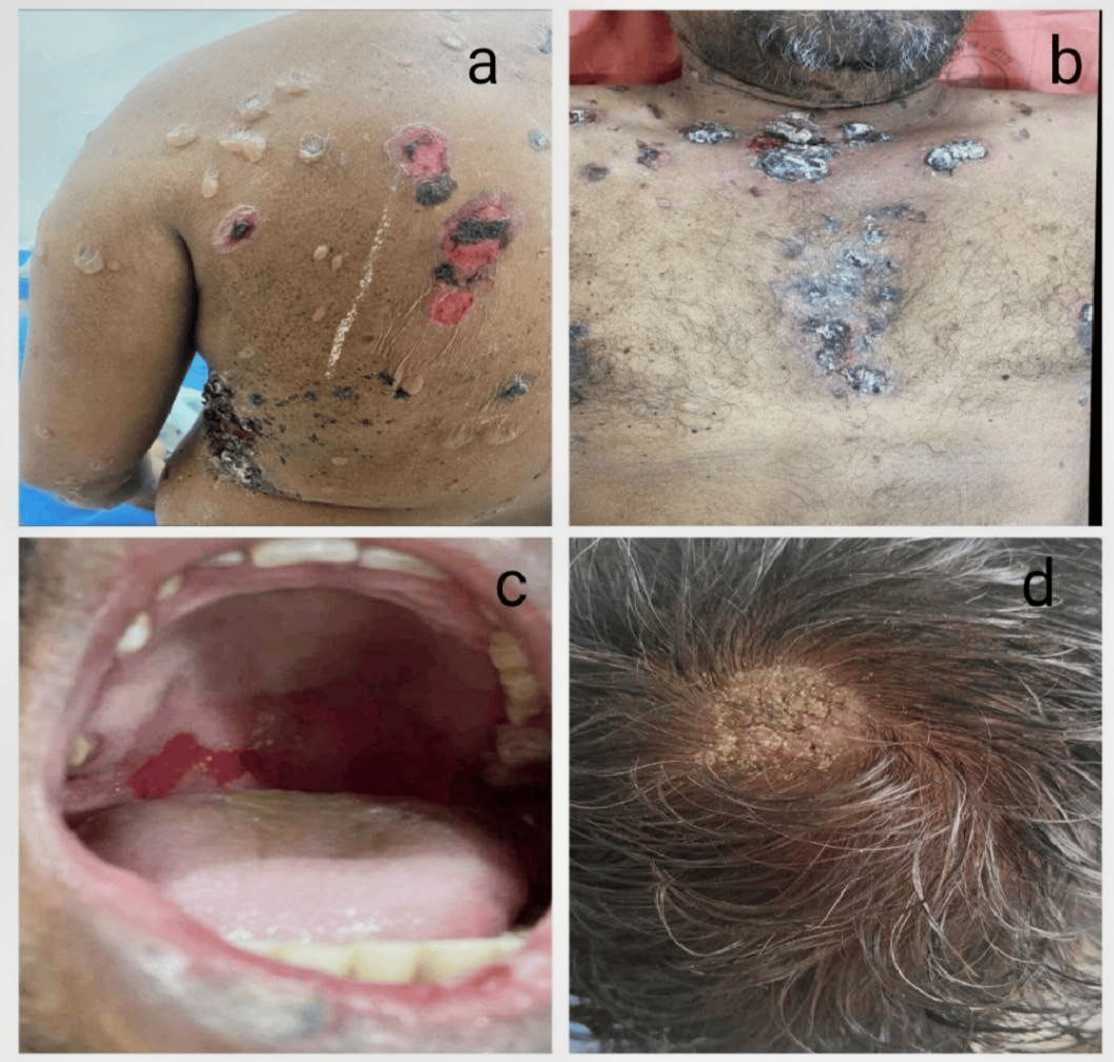
Clinical images of a case of a 45-year-old male with pemphigus vulgaris (a,b) Flaccid bullae with erosions over the trunk. (c) Oral erosions. (d) Crusted plaque over scalp.

**Figure 3:**
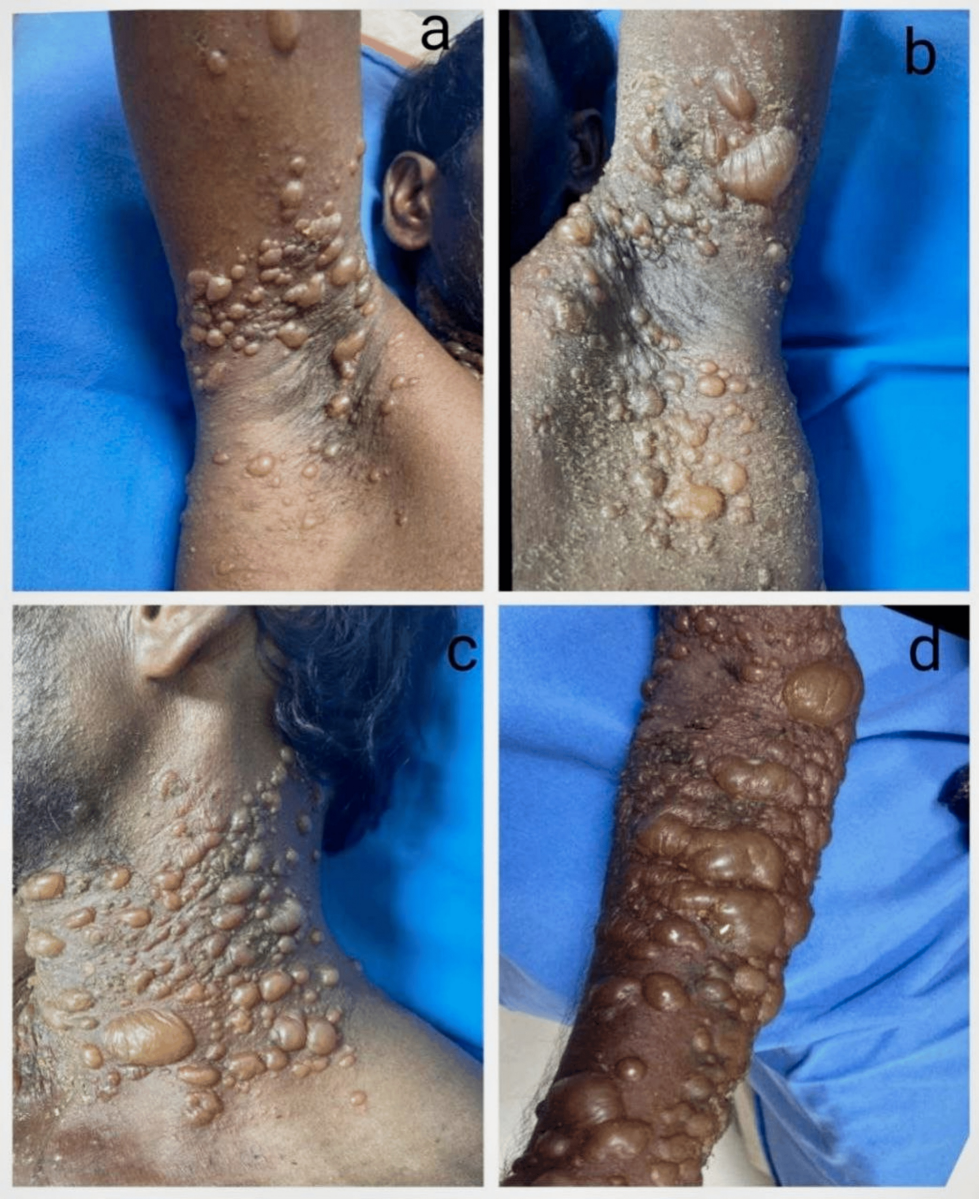
Clinical images of a 38-year-old male with bullous pemphigoid Tense bullae involving (a, b) axilla, (c) neck, and (d) forearm.

The most frequent and first site of involvement was the trunk (70%), which was followed by oral lesions, limbs, neck, and scalp (Figure 2). In a small number of instances of PV, generalized vesiculobullous lesions with a burning sensation were observed, and the majority of them healed with hyperpigmentation. The majority of cases of BP had flexural involvement, including the axilla, neck, groin, and limbs, and the lesions healed with hypopigmentation (Figure 3).

**Figure 4:**
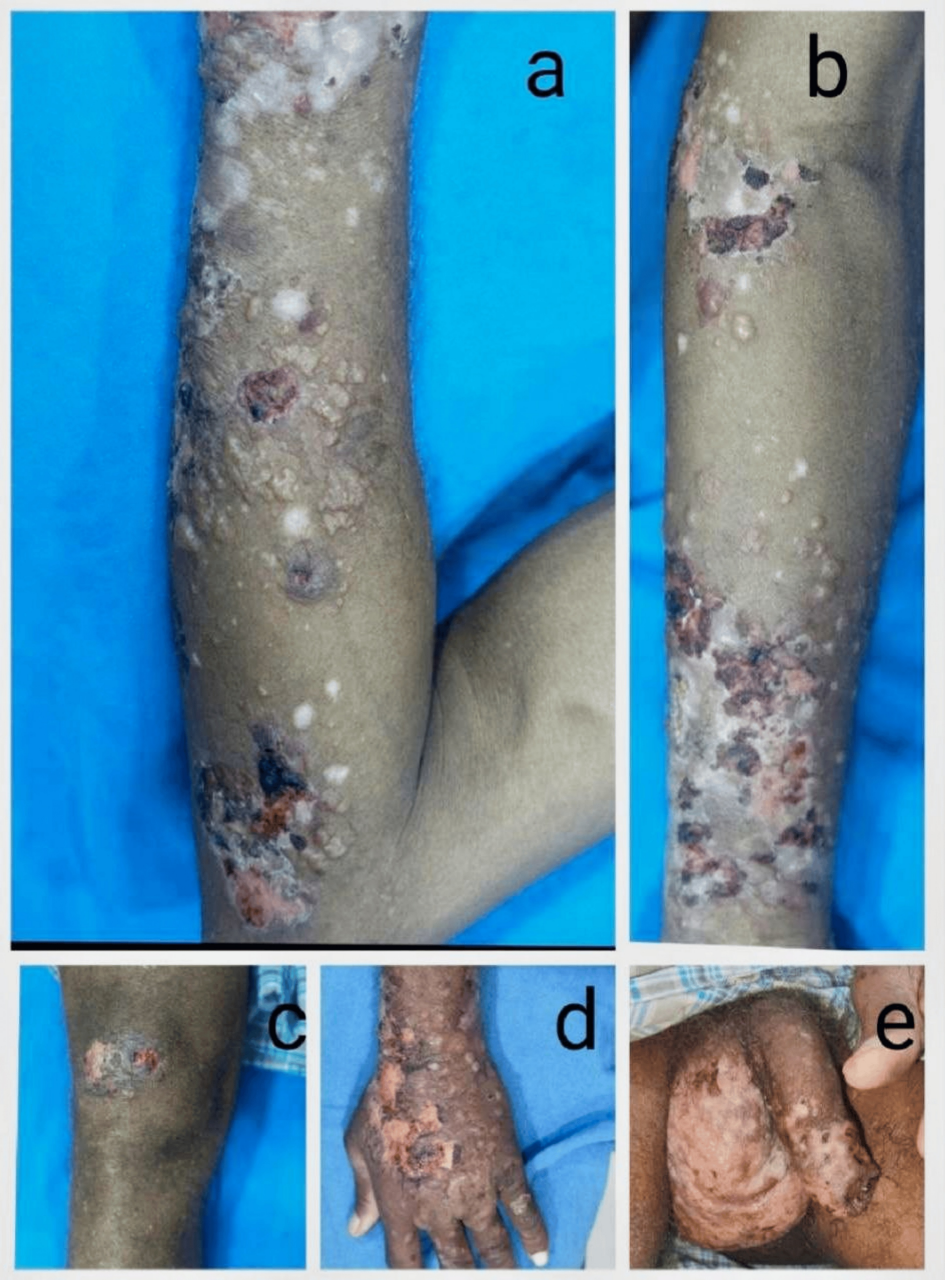
Clinical images of a 51-year-old male with epidermolysis bullosa acquisita (a-d) Tense bullae with erosions and lesions healing with hypopigmentation. (e) Erosions over scrotum, penis.

**Figure 5:**
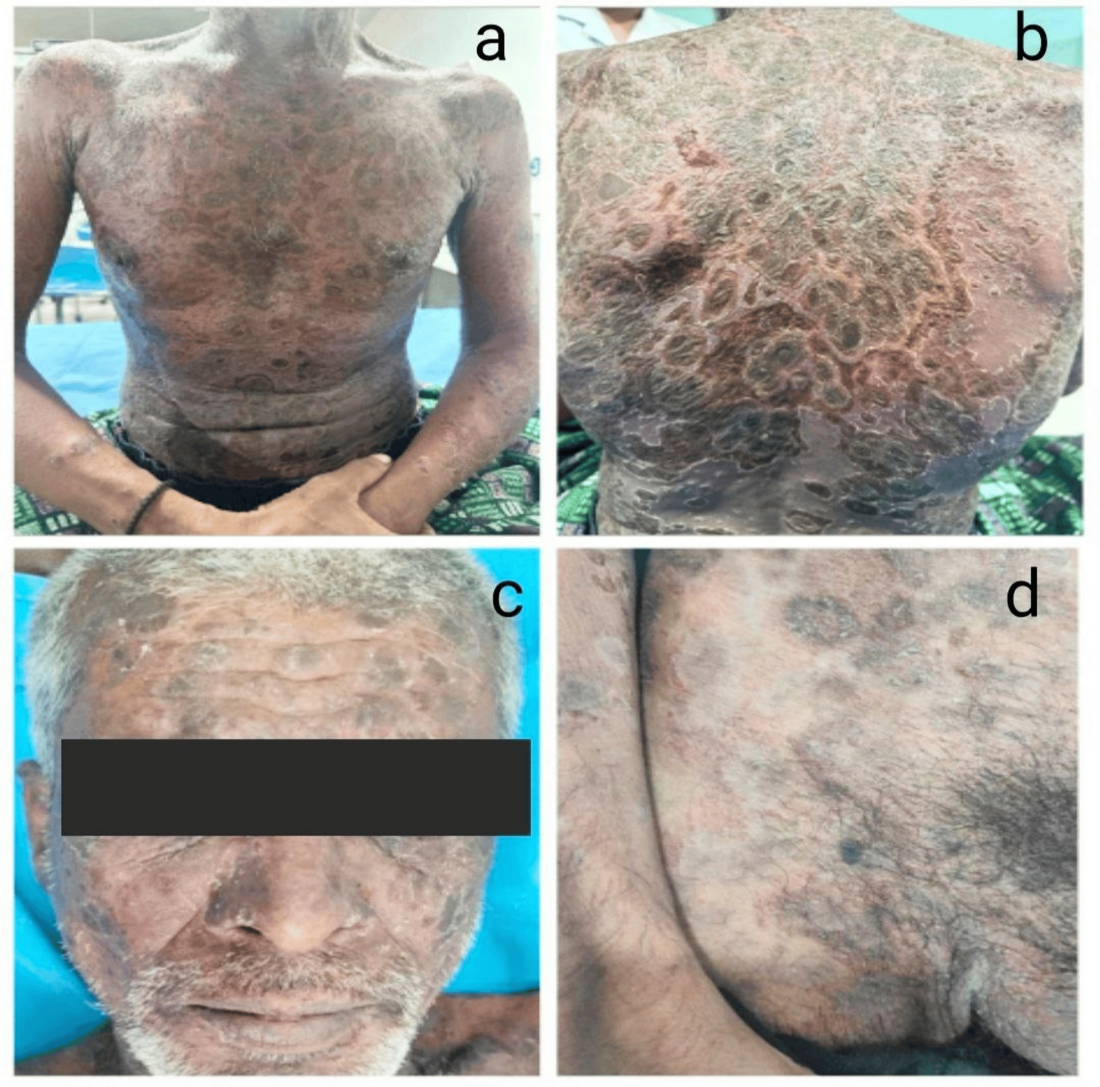
Clinical images of a 52-year-old male with pemphigus foliaceus (a, b) Crusted plaques with cornflake scaling over trunk. (c) Lesions over face. (d) Annular hyperpigmented plaques over lower trunk and groins.

**Figure 6:**
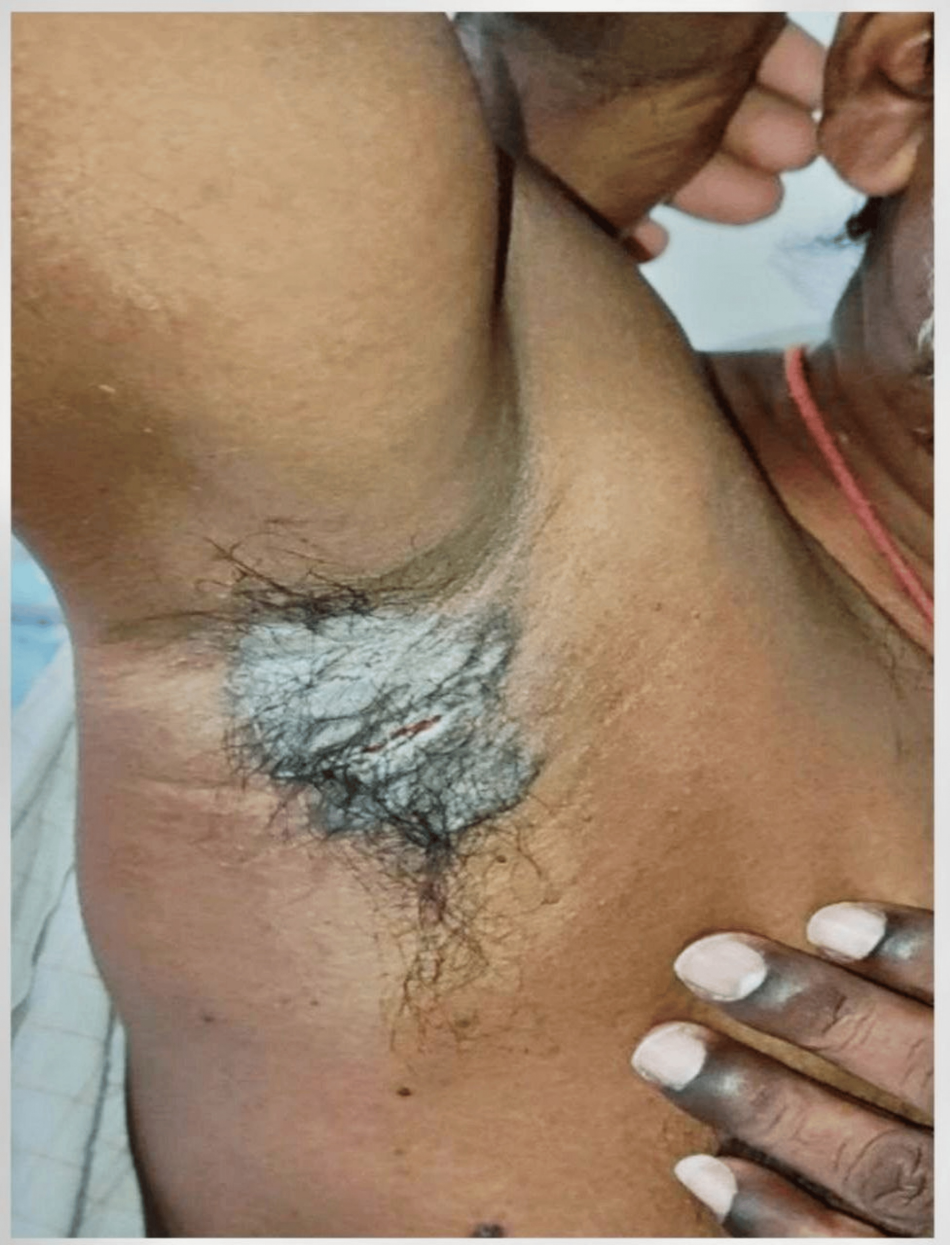
Clinical images of a 46-year-old male with pemphigus vegetans Vegetative plaques seen over axilla.

Most epidermolysis bullosa acquisita (EBA) cases involved the elbows, knees, and forearms, and the lower trunk healed with hypopigmentation (Figure 4). In one case, the lower legs were mostly involved, and there was milia. Seborrheic sites are predominantly involved with cornflake scaling in pemphigus foliaceus (PF) (Figure 5). Thick, hyperkeratotic vegetative plaques were seen in a single case of pemphigus vegetans over the bilateral axilla (Figure 6).

Bedside examinations, such as the direct and indirect Nikolsky tests, were performed with 12 cases positive for direct Nikolsky and 14 cases positive for indirect Nikolsky; the bullae spread sign was elicited in 22 cases, showing a rounded border in 10 cases, and 12 cases had an angulated border. However, the Nikolsky test is not 100% specific.

All cases underwent Tzanck smear analysis; 31 cases (62%) had acantholytic cells, 14 cases (28%) had inflammatory cells, and five cases (10%) had both inflammatory and acantholytic cells.

Mucosal involvement with oral mucosa was predominantly seen in 22 patients, and a few cases showed nasal and genital involvement (Table 5). A chi-square test performed indicates a strong association between mucosal involvement and type of disorder since the p-value < 0.05, i.e., mucosal involvement is significantly more common in pemphigus than in pemphigoid disorders (χ² = 8.84, p = 0.0029).

**Table 5:** Mucosal involvement in all cases

Mucosa		Frequency	Percentage
Involved	Oral	9	18
	Nasal	3	6
	Genital	2	4
	Oral and nasal	6	12
	Oral, nasal, genital	2	4
Not involved		28	56
Total		50	100

Distribution of cases according to the severity as per the ABSIS score system: limited pemphigus - five cases; moderate pemphigus - nine cases; significant pemphigus - 28 cases; extensive pemphigus - eight cases.

Associations

Of 50 cases, 12 patients were known cases of diabetes who are on treatment, and six cases were de novo diagnosed as diabetes; eight cases are hypertensive; four cases had cardiovascular disorder; single patient was known case of maxillary carcinoma who underwent surgery followed by chemoradiotherapy; one patient of PF had coexisting secondary syphilis.

No strong association between the presence of comorbidities and the type of autoimmune disorder was observed on performing a chi-square test since the p-value > 0.05 (χ² = 1.09, p = 0.296).

Histopathology and direct immunofluorescence

Histopathological evaluation showed 21 cases (42%) of vesiculobullous diseases with subepidermal blisters, 15 cases (30%) with suprabasal blisters, and seven cases (14%) with subcorneal blisters in the present study. In 22 cases (44%), inflammatory cell infiltrates were the most common content of the blister, with eosinophils and neutrophils being the predominant cell types. Acantholytic cells were seen in only 18% (nine) of cases. Scattered dermal, perivascular, and adnexal inflammatory infiltrates (lymphocytes) were seen in almost all the cases. DIF showed IgG and C3c deposits in a linear pattern at the dermoepidermal junction in cases of BP, and IgG and C3c deposits in a lace-like pattern in the intercellular squamous region in cases of pemphigus diseases.

Treatment

Thirty-eight of the 50 patients were initially treated with systemic steroids to manage the acute phase, and then they were maintained with topical steroids and immunomodulators like cyclophosphamide, MMF, dapsone, azathioprine, methotrexate, and rituximab. Five patients with a lesser phase of the condition were treated with topical steroids, whereas seven patients started on dexamethasone cyclophosphamide pulse (DCP) therapy. The patients’ age, comorbidities, disease kind and severity, and financial situation all played a role in the immunomodulator selection. Complete remission was observed in six cases after the stoppage of treatment for a period of two years.

Out of seven patients on DCP, three were in phase 4 with remission of disease; two in phase 3; one in the sixth cycle of phase 2, and one case in the fifth cycle of phase 1 with controlled disease.

Most of the cases (26) were in maintenance with tapering doses of systemic steroids and immunomodulators, with flare-ups in a few cases. In order to handle any side effects as soon as they occurred, two patients received two doses of inj. rituximab slow IV infusion (rheumatoid arthritis protocol) in the intensive care unit, and they responded well to the second dosage.

Patients on cyclophosphamide showed a good response, with few flare-ups throughout treatment. The majority of the time, patients' noncompliance with therapy resulted in acute flare-ups, which were managed with a brief course of systemic steroids and ongoing immunomodulators. Four cases were lost to follow-up after the acute phase was treated with systemic corticosteroids.

## Discussion

Age

Middle age (51-60 years) was the most prevalent age group, followed by the 41-50 years age group. This was comparable to a study by Alpsoy et al. in 2015 [8].

Gender

The prevailing gender in this study was female (male-to-female ratio: 1:1.27), which is consistent with a study in 2019 by Ramalingam et al. in which the male-to-female ratio was 1:1.2 [9].

Domicile

According to Chen et al. in 2024 [10], this study found that the rural (in and around Srikakulam) and low socioeconomic population, who were illiterate and primarily worked as farmers and daily wage laborers, had higher rates and severity of disease. This could be explained by a lack of medical facilities, financial constraints, and health literacy. 

Additionally, many individuals with these conditions have severe limitations in their everyday activities as a result of likely stigmatization at work; according to a study by Kianfar et al. in 2022 [11], productivity loss is not implausible.

Type

Twenty-eight of the 50 cases were intraepidermal, and 22 were subepidermal. This is comparable to a 2019 study by Ramalingam et al. [9], where the bulk of the cases were intraepidermal. 

Pemphigus vulgaris 

PV is a rare autoimmune bullous disease characterized by blistering of the skin and mucosa owing to the presence of autoantibodies against desmoglein 3 and occasionally in conjunction with desmoglein 1 [12].

The most common age of presentation for the 18 cases (male-to-female ratio: 1:1.57) in this study was 51-60 years old, which was also the result of a study conducted in 2024 by Louchez et al. [13]. Thirteen cases (72.2%) had mucosal involvement, with the oral mucosa being the first site of involvement in eleven of these cases, which is predominantly compromised, followed by lesions over the trunk and extremities. Only a small percentage of individuals had crusting, erosions, scalp lesions (33.3%), nasal, or vaginal mucosal lesions. Six individuals were receiving treatment for hypertension and diabetes.

In one case, the face, scalp, and trunk had crusted plaques and cornflake scaling with no mucosal involvement, clinically resembling PF. However, histopathological examination (HPE) suggested PV with suprabasal split, which may be interpreted as the change from PF to vulgaris, as was also noted in a 2006 study by Park et al. [14].

Five patients received DCP once a month along with routine blood pressure, liver, kidney, and sugar monitoring. Two of the patients experienced total remission for 15-18 months, and two of the patients had satisfactory disease control with one dropout.

Nine patients were receiving cyclophosphamide, and their urine was routinely checked for any alterations. After three months of treatment, one patient had red blood cells (RBCs) in their urine; the medication was stopped, and azathioprine was started for maintenance. After three months of treatment, five individuals (55.5%) had stable clinical pictures, whereas two patients did not comply with treatment. A single case that is not responding to cyclophosphamide was given inj. rituximab according to the rheumatoid arthritis protocol, disease control was seen after the two weeks of second dose. Four patients were on MMF, two had disease control, and two had a mild response with frequent recurrences as a result of medication noncompliance, which caused relapse.

Pemphigus foliaceus

PF is an autoimmune blistering disease characterized by the disruption of the epidermal cell adhesion protein desmoglein 1. PF classically presents with superficial erosions or blisters, but can rarely mimic other dermatological conditions, which makes diagnosis challenging [15]. Clinically, the majority of the nine cases with a male-to-female ratio of 1:1.25 in this investigation had flaccid bullae, cornflake scaling, and a musty odor affecting the face, neck, scalp (seborrheic sites), trunk, and limbs; two (22.2%) of these instances also had mucosal involvement. 

In a rare instance, a single patient with foliaceus and syphilis presented with hyperpigmented plaques with an annular configuration, oozing and crusting all over his body, face, and scalp. He had positive anti-desmoglein 1 and 3 levels, positive venereal disease research laboratory (VDRL) test, Treponema pallidum haemagglutination assay (TPHA), and HPE with DIF was suggestive of PF. After two months of treatment with benzathine penicillin 3 doses once per week followed by azathioprine, he showed good disease control.

After undergoing surgery and radiation therapy, a female patient with a confirmed case of carcinoma maxilla developed PF with an oral lesion within six months. The patient was put on cyclophosphamide, which helped to control the disease; a study by Luo et al. in 2024 reported that 10% of cases of cancers were linked to foliaceus [16].

Patients who adhered to long-term immunosuppressive treatment with cyclophosphamide and azathioprine showed signs of disease control. Two cases received DCP therapy; one of these cases demonstrated illness control after eight cycles in phase 1, indicating an early start to therapy because PF has a lengthy clinical course and relapsing nature that necessitates several cycles in phase 1, as reported in a study by Fontenelle et al. in 2023 [17]. Good disease control was observed in a case after two weeks of completion of the second dose of rituximab.

Pemphigus vegetans 

Pemphigus vegetans, the rarest subtype of pemphigus, is characterized by vegetative plaques, primarily affecting intertriginous areas. The most common autoantibodies target desmoglein 3 [18]. One instance of pemphigus vegetans was reported, with thick, vegetative plaques over the axilla along with oral erosions.

Bullous pemphigoid

BP is an autoimmune bullous disease characterized by sub-epidermal tense blisters, accompanied by urticarial or eczema-like lesions. Circulating autoantibodies in BP patients target BP180 and BP230 at the dermal-epidermal junction [19]. The majority of BP cases in this study occurred in people aged 55 to 65, with a male-to-female ratio of 1:1. Fifty percent of cases presented with red, itchy lesions before vesiculobullous lesions appeared, which was also seen in a study conducted in 2025 by Akbarialiabad et al. [20].

Twelve cases (85.7%) mostly involved the flexures, with other sites such as the trunk and extremities; two cases (14.2%) involved the mucosa; three cases (21.4%) appeared with large pustules, indicating subsequent infection; lesions healed with hypopigmentation.

According to a study by Castelo Branco et al. in 2022, mucous membrane involvement can happen in roughly 10-35% of patients and is nearly always restricted to the oral mucous membrane [21]; additionally, atypical non-BP should be taken into consideration as one of the possible differential diagnoses in patients who have multiple erythematous papules and nodules on their upper extremities and trunk, as reported in a 2021 study by Dbouk et al. [22].

Four of the 14 cases were diagnosed with diabetes mellitus and were using gliptins; two instances were diagnosed with chronic kidney disease and were on furosemide; these conditions may have contributed to the beginning of BP, as noted by Moro et al. in 2020 [23].

Nine of the 14 cases were begun on tab. dapsone 100 mg, three on cap. doxycycline, and two had very milder courses that were treated with topical steroids in addition to systemic steroids and IV antibiotics in patients with subsequent infections.

Epidermolysis bullosa acquisita 

EBA is a rare autoimmune disorder characterized by blistering of the skin and mucous membranes. Current pathophysiology implicates autoantibodies targeting type VII collagen, which serves as a crucial component of anchoring fibrils that attach the epidermis to the dermis [24].

All eight of the EBA cases in our investigation, with a male-to-female ratio of 1:1.6, showed tense bullae and erosions over trauma-prone sites such as the elbows, knees, flexures, trunk, and extremities. One case had oral and genital lesions, and three individuals had oral erosions. In six cases, the lesions healed with atrophic scars and hypopigmentation; these results are consistent with the case report by Szymański et al. in 2023 [25], two cases healed with milia formation. Five cases were started on systemic steroids and dapsone, which showed a good response. 

Limitations

It will be difficult to determine the illness burden throughout the entire northeast Andhra region because we have only included cases that we have seen in our tertiary care center; therefore, extensive population-based and multicentric investigations are necessary. The small sample size constrained our ability to fully adjust for potential confounders such as age, sex, comorbidities, and treatment selection bias, as it often depends on the affordability and accessibility that could have influenced outcomes independently of disease.

## Conclusions

In recent years, there has been a noticeable increase in the prevalence of autoimmune vesiculobullous illnesses. Additionally, there is a strong correlation with comorbidities such as diabetes, hypertension, cancer, and cardiovascular abnormalities, which may be brought on by prolonged steroid use. Atypical presentations should also raise suspicions, and if necessary, a biopsy must be performed to make an early and timely diagnosis. Although no significant associations were observed, further studies with larger sample sizes may be needed to better understand how demographic factors influence disease types.

To eliminate the stigma associated with the condition, people must be made aware of it and educated about the necessity of long-term therapy for early diagnosis, good compliance, and prognosis, all of which ultimately improve the patients’ quality of life.
